# Isolation, molecular characterization and sero-prevalence study of foot-and-mouth disease virus circulating in central Ethiopia

**DOI:** 10.1186/s12917-018-1429-9

**Published:** 2018-03-27

**Authors:** Mishamo Sulayeman, Fufa Dawo, Bedaso Mammo, Daniel Gizaw, Dereje Shegu

**Affiliations:** 1 Faculty of Veterinary Medicine, Hawassa University, P.O. Box 05, Hawassa, Ethiopia; 2 College of Veterinary Medicine, Addis Ababa University, P.O.Box 34, Debre Zeyit, Ethiopia; 3 National Animal Health Diagnostics and Investigation Center, P.O.Box 04, Sebeta, Ethiopia

**Keywords:** Cattle, Central Ethiopia, FMD, Molecular characterization, Risk factors, Sero-prevalence

## Abstract

**Background:**

Ethiopian livestock production and productivity is still very low due to widespread of diseases. Among the diseases, foot-and-mouth disease (FMD) is an extremely contagious and acute viral disease that causes significant economic problems in the country. A cross sectional study design was conducted from September 2015 to May 2016 to isolate and characterize FMD virus from outbreak cases; determine the sero-prevalence of antibodies against FMD virus (FMDV), and assess potential risk factors associated with sero-prevalence of the disease in selected areas of central Ethiopia. A multistage sampling technique was employed to select the study animals. Isolated viruses were characterized by antigen ELISA (IZLER, Brescia, Italy) and by genetic analysis of the sequence of the viral protein 1 (VP1). Sero-prevalence was determined using an ELISA for antibodies against non-structural proteins of FMDV based on the 3ABC proteins (ID Screen® FMD NSP Competition, ID-VET, Grabels, France). Risk factors for sero-prevalence of antibodies against FMD virus was investigated using logistic regression analysis.

**Result:**

From outbreak investigation, 28.8% (*n* = 378) cattle showed signs and lesions suggestive of FMD and 34 samples were subjected to virus isolation. Twenty eight of these cultures exhibited cytopathic effect (CPE) and were serotyped as O, A and SAT 2 FMD viruses. One A and two SAT 2 isolates named A-ETH-19-2015, SAT 2-ETH-18-2015 and SAT 2-ETH-20-2015 were further characterized by phylogenetic analysis. The overall sero-prevalence of antibodies against non-structural proteins of FMDV was 24.2% (*n* = 574). Cattle herds with crossbreed cattle, with older cattle (> 2 years), and kept together with small ruminants had higher sero-prevalences of antibodies against FMDV (*p* < 0.05).

**Conclusion:**

This study showed that FMD was present in the study areas. Among the associated risk factors, breed, age and herd composition were significantly associated with presence of antibodies against FMD virus. Three different serotypes (A, O and SAT 2) were responsible for the outbreaks of the disease. Genetic analysis indicated that the isolated viruses clustered differently from previous outbreaks. Thus, further molecular analyses coupled with protection potential of the existing vaccines against the isolates should be performed.

**Electronic supplementary material:**

The online version of this article (10.1186/s12917-018-1429-9) contains supplementary material, which is available to authorized users.

## Background

Ethiopia has huge livestock resources that play a crucial role in the livelihoods of the majority of human the population. Animal rearing is an integral part of the agricultural sector and animals represent the major drought power (95%) for crop production. The country is believed to have the largest livestock population in Africa comprising approximately 56.7 million cattle, 29.3 million sheep and 29.1 million goats [1]. The agricultural sector constitutes about 45% of the gross domestic production (GDP), more than 90% of foreign exchange earnings, 85% of employment opportunities and most of the domestic food supply [2].

Animal diseases are currently widespread in all agro-ecological zones of the country and annual mortality rates due to diseases is estimated at 8–10% for cattle herds, and 15% and 12% for sheep and goat flocks, respectively. It is estimated that animal diseases reduce the production and productivity of livestock by 50 to 60% per year [3]. Among the livestock diseases hampering productivity of the sector, foot-and-mouth disease (FMD) is considered as a bottleneck for livestock production and productivity, and is prompting trade embargos for livestock and livestock products [4].

The disease is an extremely contagious and acute viral disease of all cloven-hoofed animals and is a transboundary disease. The disease is characterized by fever, loss of appetite, salivation, vesicular eruptions in the mouth, on the feet and teats, and sudden death of young stock [5]. The recovered animals remain in poor physical condition over long periods of time leading to economic losses for livestock industries [6, 7].

FMD is caused by FMD virus (FMDV) that has seven serotypes (O, A, C, Asia 1, SAT 1, SAT 2 and SAT 3), with distinct immunologic, antigenic and genetic properties. The seven serotypes also differ in their distribution across the globe [8, 9].

Currently, four of the seven serotypes of FMDV (O, A, SAT 1, SAT 2) are endemic in Ethiopia, while serotype C was last diagnosed in 1983 [10–14]. Studies undertaken on FMD so far revealed the existence of the disease in different parts of the country, with sero-prevalence varying from 8.18% in south Omo [15] to 44.2% selected districts of Afar Pastoral Area [10].

In Ethiopia there is no government strategy to control FMD through vaccination and/or movement control. Lack of vaccination strategies (quality, coverage and timing) and presence of free animal movement without certification are thus the main factors that could increase the spread of FMD along the cattle market chain. For the development of adequate FMD control and prevention, determining the status of FMD through serological study, virus isolation and characterization of the circulating serotypes is needed. Study areas were known with the abundance of dairy farms, thus implementing both epidemiological study and outbreak investigations are essential to generate important baseline information about the disease and circulating serotypes.

Therefore, the study was designed to isolate and molecularly characterize FMDV from outbreak cases, determine the sero-prevalence of antibodies against FMDV, and assess potential risk factors associated with sero-prevalence of the disease in central Ethiopia.

## Methods

### Description of the study areas

**Fig. 1:**
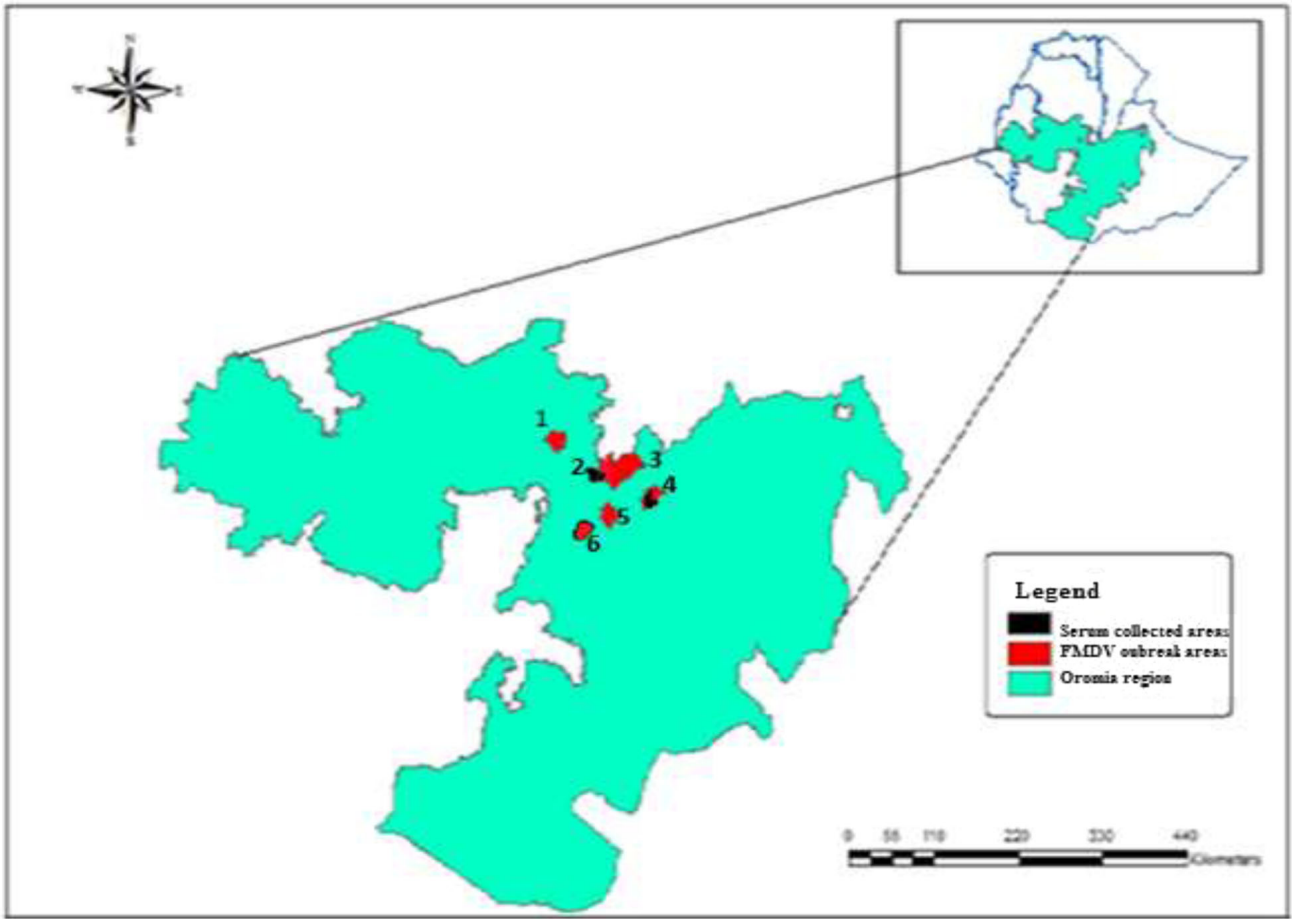
Study area

The study was conducted in three different areas, Addis Ababa (Kolfe), Arsi zone (Guna and Lude Hitosa) and East shewa zone (Adama and Boset), where FMD outbreaks occurred from September 2015 to May 2016. A cross-sectional study was also conducted to study sero-prevalence of antibodies against FMDV in and around Adama and Assela dairy farms. Detail description of the study areas with geographic and climatic parameters are presented in Table 1 and Fig. 1 [1].

**Table 1:** Description of the study areas

Study area	Altitude	Climate	Annual rainfall	Temperature	
				Maximum	Minimum
Addis Ababa	2300 m	Sub-tropical	1180 mm	22.8 °C	10.6 °C
Arsi zone	1500 to 4245 m	Sub-tropical	2400 mm	25 °C	10 °C
East shewa zone	500 to 4307 m	Tropical	410-820 mm	30 °C	18 °C

### Study animals

Outbreaks were reported by the district level animal health expertise to the regional veterinary laboratory centers. Then animals were sampled irrespective of age, breed and herd management system. Additionally, for the study of the presence of antibodies against FMDV, animals were sampled from households which keep dairy cattle, ranging from small individual farms to highly organized dairy farms.

### Study design

Cross sectional study was implemented for both outbreak investigation and sero-prevalence study of antibodies against FMDV in the study areas. Multistage sampling technique was applied. Adama and Asella districts were purposively selected sites for sero-prevalence study of antibodies against FMDV based on their accessibility, geographical location and on the abundance of dairy farms. From each district 30% of peasant associations (PAs) were selected randomly. From each PA, 20% of privately owned herds were selected randomly to obtain primary sampling unit. Finally, individual animal from each herd were selected randomly as secondary sampling unit to attain the required sample size; the strategies differed between districts as 10% of animals were sampled in Adama herds and 20% in Asella herds. Breed, age, sex, herd composition and size, and history of sampled animals were registered as to determine risk factors for the occurrence of the disease. Sampled animals were grouped into three age categories based on their dental eruption status [6].

### Sampling technique and sample size determination

Simple random sampling technique was followed to select individual animals included in the study. The sample size required for the study was calculated based on the following formula [16].

$$ \frac{\mathrm{n}=1{.96}^2\;\mathrm{X}\kern0.24em {\mathrm{P}}_{\mathrm{exp}}\mathrm{X}\ \left(1-{\mathrm{P}}_{\mathrm{exp}}\right)}{{\mathrm{d}}^2} $$

Where, n = sample size, P_exp_ = expected prevalence, and d = absolute precision.

The expected prevalence considered was 14.5% for Adama based on a previous study in the area [9], while 50% was used in Asella due to lack of previous studies. Accordingly with 95% confidence level, 5% absolute precision and expected prevalence as mentioned above, the sample size computed was 190 for Adama and 384 for Asella. Thus, the total sample size required for the study was 574 dairy cattle.

### Sample collection

#### Tissue and oro-pharyngeal (OP) fluid samples

Epithelial tissue samples were collected from unruptured and freshly ruptured vesicles and kept in 0.04 M phosphate buffer with 50% glycerol [17]. Samples of OP fluid were collected, from apparently healthy cattle that were found in close proximity to the affected herds, by a probang cup and poured into a 20 ml bottle. Fluid samples containing cellular material was added to 5 ml tube containing about 2 ml of transport medium at sites where the samples were collected [17]. Then, the samples were labeled and kept at cold chain during transportation to NAHDIC, Sebeta, where they were kept at -80 °C until processed [18].

#### Blood

From each animal, 10 ml of blood was collected and kept at room temperature overnight. Then serum was separated and transferred into cryovial at Asella Regional Veterinary Laboratory and stored at − 20 °C. At the end of sampling sera were transported in cold chain to NAHDIC, Sebeta, and stored at − 20 °C [17].

### FMD virus isolation

Tissue samples were ground in a small volume of tissue culture medium containing antibiotic (penicillin [1000 International Units (IU)], neomycin sulphate [100 IU], polymyxin B sulphate [50 IU], mycostatin [100 IU]) using sterile sand, pestle and mortar, and diluted in the same medium to a 10% suspension [18], which was centrifuged at 2000 rpm for 10 min and filtered through a membrane filter with a 0.22 μm pore size. About 1 ml of filtered tissue suspension was inoculated on confluent baby hamster kidney (BHK-21) (AU/PANVAC) monolayer cells grown in 25cm^2^ tissue culture flask and incubated at 37 °C for 1 h for adsorption of the virus. Then cell cultures were added 8 ml of maintenance medium (2% MEM) and incubated at 37 °C and 5% CO2 in a humidified incubator. Cell cultures were examined for evidence of cytophatc effect (CPE) every 24 h for a maximum of 72 h, and CPE-positive cell cultures harvested at 85% CPE. Cell cultures without CPE after 72 h were passaged harvested and passaged onto new monolayers as described above. Samples that did not develop CPE during the two passages were considered virus negative [18, 19].

### Serological diagnostic tests

The collected sera were tested by FMDV 3ABC-Ab ELISA (ID Screen® FMD NSP Competition, ID-VET, Grabels, France) for the detection of antibody to 3ABC polyprotein which is a useful indicator of past FMDV infection regardless of the serotype involved (Additional file 1).

### Antigen detection ELISA

Sandwich ELISA (IZLER, Brescia, Italy) was performed on isolates with selected combinations of anti-FMDV monoclonal antibodies (MAbs). The kit was designed for serotyping of FMDV (Additional file 2).

### Reverse transcription polymerase chain reaction (RT-PCR) and sequencing

CPE-positive cell culture harvests were examined for FMDV using RT-PCR and primers that amplify VP1 of FMDV and selected, representative VP1s were sequenced. All RNA extraction, RT-PCR, and sequencing were conducted in WRL for FMD, Pirbright Institute, UK [20].

### Molecular evolutionary relationship

A total of one VP1 sequence of serotype A FMDV and two sequences for serotype SAT 2 FMDV were obtained, and one for each of these serotypes used for online blast search to retrieve closely related sequences from Genbank using Molecular Evolutionary Genetic Analysis software (MEGA, V 6.06) [20].

### Ethical approval

Ethical approval and consent for this study was obtained from Addis Ababa University College of Veterinary Medicine and Agriculture Minutes of Animal Research Ethics and Review committee (Reference AREC001/2016). Verbal consent was also obtained from the farm managers to take samples from their cattle and for further research use of the samples.

### Data management and analysis

The VP1 nucleotide sequences were aligned by using Clustal W program imbedded in the MEGA software. The alignment sequences were used to construct phylogenetic tree analysis using midpoint-rooted neighbor-joining tree and Kimura 2-parameter nucleotide substitution model using MEGA 6.06. The robustness of the tree topology was assessed with 1000 bootstrap replicates as implemented in the program.

Data generated from laboratory investigations were recorded and coded using Microsoft Excel spreadsheet (Microsoft Corporation) and analyzed using STATA version 11.0 for Windows (Stata Corp. College Station, TX, USA).

The Odds ratio (OR) was calculated for each risk factor for sero-positivity to FMD. In all the analyses, confidence levels at 95% were calculated, and a *P* < 0.05 was used for statistical significance level. The OR was calculated for the risk factors and sero-positivity of the disease to determine the degree of association between risk factors and the disease. Descriptive statistics like prevalence was used to calculate sero-positivity by dividing the number of FMDV antibodies positive animals by the total number of animals tested.

## Results

### Clinical examination of FMD outbreak

From outbreak investigation, 28.8% (*n* = 378) cattle showed signs and lesions suggestive of FMDV infection. The major important clinical signs observed during the outbreak investigation were salivation and lameness. Mouth lesions consisted of erosions and ulcers on tongue and dental pad, whereas foot lesions comprised erosions on the inter-digital space and the coronary bands. In severe cases the hooves of affected animals tended to separate from the coronary bands. Reluctant to move and lagging behind herds and refusal of grazing were also seen as common features of clinically affected animals.

### FMD virus isolation and characterization

A total of 30 epithelial tissue and 4 OP fluid samples collected from the three study sites, were subjected to virus isolation using BHK21 cell culture. CPE characterized by a fast destruction of BHK-21 monolayer cell within 48 h was registered for 28 tissue samples only.

### FMDV serotype identification and distribution

Three serotypes namely serotype A, O and SAT 2 were identified from the outbreak samples (Table 2). Different outbreaks were caused by different serotypes: in Arsi zone only serotype A was responsible for the outbreak while in East Shewa zone only serotype SAT 2 was responsible. Surprisingly, in Addis Ababa three serotypes (A, O and SAT 2) were responsible for the outbreaks observed during the study.

**Table 2:** FMDV serotypes and their topotype identified

Site of outbreaks			No. of samples	CPE positive	Serotype		Topotype
					Sandwich ELISA result (positive)	RT-PCR Result	
Region	Zone	District					
Oromia	Arsi	Guna	12	8	A (8)	GD^a^	Africa
		Ludehitosa	6	5	SAT 2 (5)	GD^a^	VII
	East shewa	Adama	4	4	SAT 2 (4)	GD^a^	VII
		Boset	5	4	SAT 2 (4)	NT	NT
Addis Ababa	Addis Ababa	Kolfe	7	7	O (3), A (2) & SAT 2 (2)	NT	NT
Total			34	28			

*CPE* Cytopathic effect, *GD* Genome detected, *ELISA* Enzyme Linked Immuno-Sorbent Assay, *NT* Not tested, *RT-PCR* Reverse transcription polymerase chain reaction

^a^ One representative isolate sequenced

### Molecular characterization (phylogenetic analysis)

**Fig. 2:**
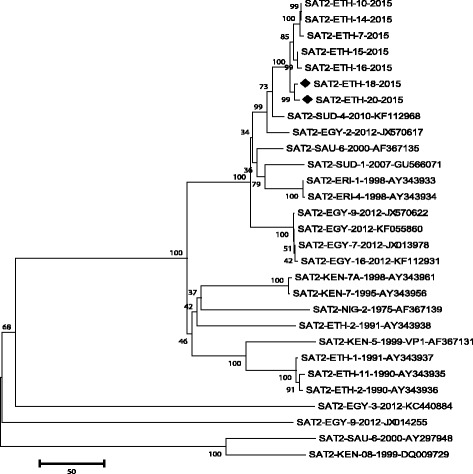
Serotype SAT 2 phylogenetic tree

Isolated viruses SAT2/ETH/18/2015 from Adama and SAT2/ETH/20/2015 from Ludehitosa districts were compared based on 648 nucleotide sequence of VP1. The viruses shared 99.07% genetic similarity with each other, and > 90% genetic similarity with three other SAT 2 FMDV isolates from Ethiopia (SAT2/ETH/15/2015, SAT2/ETH/10/2015 and SAT2/ETH/14/2015 from Sidama (SNNPR), Awi (Amahara) and North Shoa (Oromia), respectively. These five SAT 2 FMDVs were homologous, geographically clustered and formed a single genetic lineage called topotype VII and genotype Alx-12. The genetic relationship of the isolates with the other SAT 2 serotypes is displayed on phylogenetic tree (Fig. 2).

**Fig. 3:**
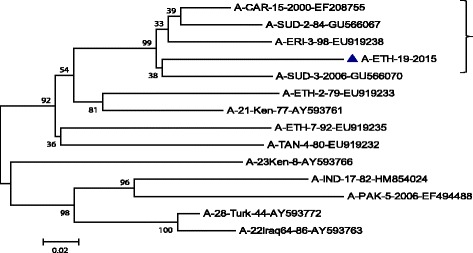
Serotype A phylogenetic tree

One representative serotype A isolated from Guna District, Arsi zone of Oromia region, during the study period was also compared with other countries FMD serotype A isolate sequences. The isolated serotype A in the current study falls into African topotype and genotype IV. The genetic relationship of the isolates with the other A serotypes is displayed on phylogenetic tree (Fig. 3).

### Sero-prevalence of antibodies against FMDV

From the total of 574 sera collected from dairy animals and tested by 3ABC-Ab ELISA, 24.22% (*n* = 139) were found positive. The higher sero-prevalence was observed in and around Adama town compared to Asella (Table 3) but the difference was not statistically significant (*p* > 0.05).

**Table 3:** Sero-prevalence of antibodies against FMDV in dairy cattle in and around Adama and Asella towns

Origin	Sera tested	Test (+) (%)	95%CI	χ^2^	*p*-value
Adama	190	51 (26.8)	0.20–0.33	1.06	0.304
Asella	384	88 (22.9)	0.18–0.27		
Total	574	139 (24.2)	0.20–0.27		

(+) Positive, *CI* Confidence Interval

### Sero-prevalence in relation to host intrinsic risk factors

#### Sero-prevalence of antibodies against FMDV among different age groups

**Fig. 4:**
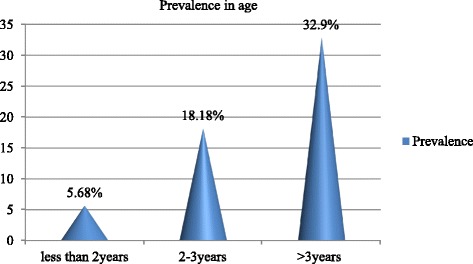
Sero-prevalence of antibodies against FMDV among different age groups in dairy cattle in and around Adama and Asella towns

Sero-prevalence of antibodies against FMDV were compared berween different age groups of dairy cattle. An increasing sero-prevalence trend was observed with increasing age (Fig. 4) and the difference was statistically significant among age groups (χ2 = 37.43; *P* < 0.001).

#### Sero-prevalence of antibodies against FMDV between sex groups

The sero-prevalence of antibodies against FMDV was 25.26% in females and 18.95% in males (Table 4), but this difference was not statistically significant (*P* > 0.05).

**Table 4:** Sero-prevalence of antibodies against FMDV between sex groups in dairy attle in and around Adama and Asella towns

Sex	Sera tested	Test (+) (%)	95% CI	χ2	*p*-value
Female	479	121 (25.3)	0.21–0.29	1.80	0.179
Male	95	18 (19)	0.09–0.38		
Total	574	139 (24.1)	0.21–0.28		

(+) Positive, *CI* Confidence Interval

#### Sero-prevalence of antibodies against FMDV in different cattle breeds

Interestingly, sero-prevalence of antibodies against FMDV differed significantly (χ2 = 14.02; *P* < 0.05) between local (12.69%, *n* = 134) and crossbred (27,73%, *n* = 440) cattle.

### Sero-prevalence of antibodies against FMDV in relation to host extrinsic risk factors

#### Sero-prevalence of antibodies against FMDV in relation to herd size

Sero-positivity and herd size seems to have positive relationship in that an increasing sero-prevalence of antibodies against FMDV was observed as herd size increases (Table 5); but the difference was not statistically significant (*P* > 0.05).

**Table 5:** Sero-prevalence of antibodies against FMDV in relation to herd size in dairy cattle in and around Adama and Asella towns

Herd size	Sera tested	Test (+) (%)	95%CI	χ2	*p*-value
Small	438	98 (22.4)	0.18–0.26	4.76	0.092
Medium	77	20 (26)	0.16–0.36		
Large	59	21 (35.6)	0.23–0.48		

(+) Positive, *CI* Confidence Interval

#### Sero-prevalence of antibodies against FMDV in relation to animal composition and management system

Significantly higher sero-prevalence of antibodies against FMDV was recorded in cattle kept together with small ruminants than those that were not (*P* < 0.05) (Table 6). Animals managed semi-intensively were more likely to have antibodies against FMDV than animals under intensive management (Table 6) but the prevalence was not significantly different (*P* > 0.05).

**Table 6:** Sero-prevalence of antibodies against FMDV in relation to animal composition and management in dairy cattle in and around Adama and Asella towns

Risk factors	Tested	Test (+) (%)	95% CI	χ2	*p*-value
Animal composition					
Only cattle	422	93 (22)	0.18–0.26	3.99	0.040
Cattle and small ruminants	152	46 (30.7)	0.23–0.38		
Management					
Intensive	254	55 (21.7)	0.17–0.27		
Semi-intensive	320	84 (26.3)	0.21–0.31	1.64	0.200

(+) Positive, *CI* Confidence Interval

### Logistic regression analysis

The univariable and multivariable logistic regression analysis included effects of breed, history of herd contact with small ruminants, and age of animals (Tables 7 and 8). Univariable logistic regression revealed cross-bred cattle were 2.64 times more likely to have antibodies against FMDV than local breeds. The risk of having antibodies against FMDV was increased (odds ratio (OR) = 1.54) when herds of cattle and small ruminant were kept together compared to herd of cattle kept alone. Animals greater than 3 years old were found 8.14 times more likely to be sero-positive of antibodies against FMDV than young animals (those found below 2 years old).

**Table 7:** Univariable logistic regression analysis of potential FMD risk factors in dairy cattle in and around Adama and Asella towns

Explanatory variable	Variable categories	OR	95% CI	*P*-value
Breed	Cross vs. local	2.64	1.52–6.67	0.001
Cattle composition	Kept with small ruminants vs. without	1.54	1.01–2.33	0.046
Age	Old vs. young	8.14	3.20–20.70	0.000
	Adult vs. young	3.69	1.38–9.83	0.009

*OR* odds ratio, vs. versus, *CI* confidence; old > 3 years, adult 2–3 years, young < 2 years old

**Table 8:** Multi-variable logistic regression analysis of potential risk factors for dairy cattle sero-positivity in and around Adama and Asella towns

Explanatory variable	OR	SE	95% CI	*P*-value
Breed	2.79	0.81	1.59–4.92	0.000
Animal composition	1.54	0.34	0.99–2.38	0.048
Age	2.55	0.44	1.81–3.58	0.000

*OR* odds ratio, *SE* standard error, *CI* confidence interval

## Discussions

During outbreak investigation, 28.8% (*n* = 378) cattle showed signs and lesions suggestive of FMD. In agreement with this finding, Negussie et al. [21] reported 28.2% clinically sick animals after conducting a number of outbreak investigations in different parts of the country.

In current study, three serotypes (A, O, and SAT 2) of FMD viruses were isolated. Serotype O was isolated from the samples collected from Kolfe district (Addis Ababa). This result is in line with previous serotype O FMDV outbreaks in Ethiopia [9, 13] and the most prevalent serotype worldwide [22].

Serotype A was isolated from the samples collected from Kolfe and Guna district of Addis Ababa and Arsi zone, respectively. Previously serotype A was reported from bovine samples collected from Hadiya and Yabello areas [23, 24]; similarly from samples collected from different outbreak areas of Ethiopia [24].

Serotype A identified from Guna District was clustered to genotype IV of African topotype. This genotype was not reported before from this area and more detailed analysis need to be conducted using advanced software’s. This is in support of the previous findings that reported serotype A FMDV belonged to African topotype [19] and all viruses from Ethiopia belonged to the African topotype [25].

Serotype SAT 2 FMD viruses were identified from cattle found in Ludehitosa district (Arsi zone), Adama and Boset district (East shewa zone) and Kolfe district (Addis Ababa). Previously serotype SAT 2 was isolated and reported from cattle in Addis Ababa [9], Borana pastoral area [13] and Benshangul-Gumz [9]. Serotype SAT 2 FMDV has previously been reported from many sub-Saharan African countries [26–28] suggesting endemicity of the serotype in these countries.

The presently identified SAT 2 serotype was clustered to topotype VII. This is in agreement with the findings of [9], who reported that SAT 2 viruses circulating in Ethiopia belonged to topotype VII along with the Sudanese isolates. This indicate that SAT 2 was introduced to the present study districts; the possible way of entry of the virus might be related to communal resource utilization and free movement of animals across the regions because SAT 2 is endemic to different regions of the country [9, 13] and neighboring African countries like Sudan republic [18, 29, 30]. The present isolates seem to be different from previously published isolates as they cluster differently on the tree. Further detailed genetic analysis like genetic distance determination is needed to have more insight about these viruses.

The FMDVs causing 2015 outbreaks in Ethiopia were not closely related to previous Ethiopian isolates. These new introductions are likely to have happened through uncontrolled trans-boundary movements of animals, which constitute a significant risk for virus crossing the border in both directions. This is due to lack of strong animal movement regulation across the border and the ability of the virus to transmit with the wind. This explanation is supported by a previous study which conclude closely related viruses could either be from the same outbreak or from the other oubreaks due to closely related viruses [29].

The overall sero-prevalence of antibodies against FMDV was 24.22%, which agreed with the overall sero-prevalence of 26.5% found in an Ethiopian pastoral area [12]. Compared to the present findings lower prevalence of 5.6% [21], 8.01% [31] and 9% [23] were reported from different parts of Ethiopia. On the other hand relatively higher sero-prevalence was previously reported from Eastern zone of Tigray with 41.5%; followed by the Guji zone of Oromia and Yeka district of Addis Ababa city, with 32.7% and 30%, respectively [25].

The sero-prevalence of antibodies against FMDV was significantly higher when cattle and small ruminants were kept together. Previous study conducted in four selected Districts of Gambella Regional State reported a similar effect [23]. In Sub-Saharan Africa [30], similar high sero-prevalence of FMD where communal farming of cattle, sheep and goats occur, indicated a high sero-prevalence of antibodies against the virus infection associated with sheep and goats in the absence of clinical signs. This suggests that small ruminants have an important role in the epidemiology of FMD as they can serve as potential carriers and transmitters of the disease [32].

Sero-prevalence of antibodies against FMDV increased with increasing herd size. This may be attributed to crowding of animals that can facilitate frequency of direct contact and hence enhances chances of transmission. A study conducted around South Omo Zone and Borana pastoral area reported a similar effect [15, 33].

Significantly higher sero-prevalence of antibodies against FMDV in old and adult animals than in young observed in the current study was in agreement with the previous reports of [8] in Borena pastoral area and [33] Awbere and Babille districts of Jijiga zone. This effect can be a result of cumulative exposures over time, but is also to some extent caused by the persistence of antibodies against FMDV for extended periods of time [27]. Aged animals might have acquired the infection from multiple serotypes, and could have produced antibodies against those serotypes. Relatively low sero-prevalence in animal groups below 2 years old might be indicative of the existence of passive maternal immunity and low frequency of exposure [10, 33].

Higher sero-prevalence of antibodies against FMDV in cross-bred cattle might be associated with the genetic difference among cattle breeds. These indicated that cross-breeds may be more susceptible to FMDV endemic in Ethiopia, as is also reported Sub-Saharan Africa [34].

The presence of this disease in the country is a major obstacle to the development of livestock resource because of its adverse effects on production and their product exports. In Ethiopia, factors such as the presence of high numbers of susceptible domestic animals, herd composition or the involvement of multiple hosts (cattle, sheep and goats), herd size and individual animal age variability, lack of prophylactic vaccination, absence of regulation for prohibition of animal movement, high contact of animals at marketing and common grazing place as well as at watering points could contribute to the occurrence of FMD and create the difficulty in controlling the outbreaks.

## Conclusion

FMD is prevalent in the study Districts as shown clinically, serologically, virologically and by molecular characterization and reported to be endemic in Ethiopia. During the study period serotype A, O and SAT 2 were identified, with highest prevalence of serotype SAT 2. Serotype SAT 2 and A isolated in Ethiopia were genotype Alx-12, topotype VII and genotype IV, African topotype, respectively. But these molecularly characterized serotypes seem to be emerging viruses as they cluster differently from the previously reported FMD viruses from Ethiopia.

An extensive regular surveillance, serotyping of the outbreak isolates, regular vaccination and movement control should be implemented. Also it is important to continue characterization of circulating FMDV and to match field isolates to vaccine strains to support the control of the disease by vaccination.

## Additional files

Additional file 1: Serological diagnostic tests procedures. Procedures and principles of the test during the study were discussed in detail. (DOCX 12 kb)

Additional file 2: Antigen detection ELISA procedures. Antigen detection ELISA procedures and result interpretation during the study were discussed in detail. (DOCX 13 kb)

## Abbreviations

BM: Bedaso Mammo
CI: Confidence interval
CPE: Cytopathic effect
DG: Daniel Gizaw
DS: Dereje Shegu
ELISA: Enzyme-linked immunosorbent assay
FD: Fufa Dawo
FMD: Foot-and-mouth disease
FMDV: Foot-and-mouth disease virus
GDP: Gross domestic production
MAb: Monoclonal antibodies
MS: Mishamo Sulayeman
NAHDIC: National animal health diagnostic and investigation centre
OR: Odd ratio
PA: Peasant association
PA: Peasant association
PBS: Phosphate-buffered saline
SAT: Southern African territories
WRL: World reference laboratory
