# Supplementary material for: Isolation, molecular characterization and sero-prevalence study of foot-and-mouth disease virus circulating in central Ethiopia
Source: BMC Vet Res. 2018 Mar 27;14:110. doi: 10.1186/s12917-018-1429-9 (PMC5870258; doi:10.1186/s12917-018-1429-9)
Supplement: Supplementary file 1 — Serological diagnostic tests procedures. Procedures and principles of the test during the study were discussed in detail. (DOCX 12 kb) [file 12917_2018_1429_MOESM1_ESM.docx]

### Additional file 1: Serological diagnostic tests procedures

Briefly, the test was carried out stepwise as per the manufacturer’s manual. First all reagents were kept at room temperature and homogenized by vortexing. The test was carried out in 96 well micro plates. Then 50μl of dilution buffer 18 were added in to each well. Thirty μl of positive control were added in to wells A1 and B1 and the same volume of negative control were also added to wells C1 and D1 while, the rest wells were filled by 30μl of test sera. Then incubated at 37^o^C for 2hours, after incubation the wells were washed 5 times with adding 300μl of wash solution. After washing 100μl of the conjugate 1X were added in to each wells and incubated for 30min at 21^o^C. After incubation the wells were washed 5 times with 300μl of wash solution, then 100μl of the substrate solution (TMB) was added in to each wells and incubated at 21^o^C for 15minutes in dark. To stop color reaction 100μl of stop solution was dispensed into each well. Finally the optical density (OD) readings were recorded using a spectrophotometer at wavelength of 450nm.

Validation

The test result was validated if:

- The mean value of negative control O.D. (OD_NC_) was greater than 0.7.

OD_NC_ > 0.7

- The mean value of the positive control O.D. (OD_PC_) is less than 30% of the OD_NC_

OD_PC/_OD_NC_ <0.3

Interpretation

For each serum sample, the competition percentage was calculated (S/N %):

S/N % = OD _sample_/OD_NC_ X 100

Sample presented S/N %:

- Less than or equal to 50% were considered positive
- Greater than 50% were considered negative
