# Supplementary material for: Isolation, molecular characterization and sero-prevalence study of foot-and-mouth disease virus circulating in central Ethiopia
Source: BMC Vet Res. 2018 Mar 27;14:110. doi: 10.1186/s12917-018-1429-9 (PMC5870258; doi:10.1186/s12917-018-1429-9)
Supplement: Supplementary file 2 — Antigen detection ELISA procedures. Antigen detection ELISA procedures and result interpretation during the study were discussed in detail. (DOCX 13 kb) [file 12917_2018_1429_MOESM2_ESM.docx]

### Additional file 2: Antigen detection ELISA procedures

The test was conducted as per the manufacturer’s recommendation. Since 6 samples were feasible to test on a micro plate containing 96 wells, one positive control for each of FMD types O, A, SAT 1 and SAT 2 and negative control were included in each plate. These controls were already incorporated into the ELISA micro plate (trapped by the respective catching MAb). First samples were diluted ½ in diluent buffer and 50μl of each diluted sample was distributed in 72 wells of A-F rows: two replicates of each-specific catching MAb and for the pan-FMDV MAb. Then, 50μl of diluents per well were added in all wells of G and H (positive and negative control respectively) rows; then plates were incubated at 25^o^C for 1hour. After incubation, all fluids on the plates were discarded and all remaining residual fluids were removed by tapping. Then 200μl of washing solution were added and incubated for 3min at room temperature, subsequently wells were emptied and the washing repeated twice (three washing cycles in total). Then all residual fluids were removed by tapping on clean absorbent paper and 50μl of conjugate A were added from columns 1 to 8 and the same volume of conjugate B were added from columns 9 to 12. Plates were covered and incubated at room temperature for 1hour. After incubation 50μl of substrate (TMB) per well were added to all wells and plates were covered and left at room temperature for 20minutes in the dark. Reaction was stopped by adding 50μl of stop solution (sulfuric acid (H_2_SO_4_)). Immediately after blocking, reading the optical density (OD) of each well was done at 450 nm wavelength using micro plate reader.

Criteria for test validity

The positive controls were expected to give OD values of 1.0 unit or higher in the type-specific reactions and in the pan-FMDV reaction, the negative control usually gives OD values lower than 0.1 in wells H1 to H8 and slightly higher in wells H9 to H12.

Table 1: Interpretation of OD values for antigen detection ELISA

| Negative for FMDV | OD <0.1 with all catching MAbs, after subtracting the OD of the respective negative control. |
| --- | --- |
| FMDV positive type O | OD ≥ 0.1 with the type O MAbs and the pan-FMDV O, A, C, Asia 1 MAb. Some samples may cross react with MAb A 4D12, but OD values wit MAb O are higher. |
| FMDV positive type A | OD ≥ 0.1 with at least one of the two type A MAbs and with the pan-FMDV O,A,C,Asia1 MAb |
| FMDV positive type SAT1 | OD ≥ 0.1 with the type SAT1 catching MAbs, after subtracting the OD of the respective negative control |
| FMDV positive type SAT2 | OD ≥ 0.1 with the type SAT2 catching MAbs, after subtracting the OD of the respective negative control |
| FMDV positive (untyped) | OD ≥ 0.1 with the pan-FMDV MAb and <0.1 with the type-specific MAbs, after subtracting the OD of the respective negative control |
